# Supplementary material for: Prevalence of depression among students at Ethiopian universities and associated factors: A systematic review and meta-analysis
Source: PLoS One. 2023 Oct 12;18(10):e0288597. doi: 10.1371/journal.pone.0288597 (PMC10569578; doi:10.1371/journal.pone.0288597)
Supplement: Attachment — Submitted filename: Comments to the authors.docx [file pone.0288597.s003.docx]

**Comments to the authors**

PLOS ONE

PONE-D-22-34689

Article Type: Study Protocol

Prevalence and associated factors of depressive symptoms among Ethiopian University students: a systematic review and meta-analysis

First of all, I would like to thank the journal’s editor(s) for believing in me to review this work. I would also like to thank the authors for conducting such a review. However, the work has several minor flaws, which are provided below.

**Title and abstract**

First and foremost, is your work a “protocol” or a “full review”?

As can be easily inferred from the title, the authors tried to review the prevalence of the symptoms of depression. However, they did not show which symptom of depression is the most prevalent, and so on. Unless the title should be rephrased as “Prevalence of depression among students at Ethiopian universities and associated factors: a systematic review and meta-analysis.”

Avoid saying “Ethiopian university students,” which implies that only students with Ethiopian citizenship. It might be more acceptable to say "students at Ethiopian universities." Because, grammatically, it makes sense, but its meaning deviates from the aim. Or if the authors' intention was to address only Ethiopian students, they would be right.

“The pooled prevalence of depressive symptoms was 28.13% (95% CI: 22.67, 33.59).” But where are the symptoms? In fact, symptoms, which are only expressed by the person with the condition of interest, are indicators of a disorder. However, the symptoms should be listed in order of their prevalence, as per the aim of the review. We know that depression has various symptoms. This pooled data only showed the prevalence of depression, whether major or minor. The conclusion is ambiguous.

“…one-fourth of students in Ethipisn University…” But the title is different; it is about “Ethiopian university students," not “students in Ethiopian universities.” It is also good to revise grammar issues throughout the manuscript. For instance, because the study is a review covering various universities, it is better to write "universities" than "university." See also the word "Ethipisn" in this phrase.

**Background**

Page 3 and 4, lines 70-90: This should be discussed in comparing with your finding under the discussion section. In the introduction section, it is better to explain and discuss the extent to which depression impacts the life and activities of students in universities.

Page 3, line 73: “…42.66 of students…” What did you mean?

Page 3, line 82: “The most consistently significantly associated factors among university students…” What did you mean? Please revise grammar issues throughout the manuscript.

**Materials and methods**

Page 5, lines 110-112: “Our search was conducted on the 10th of the October 2022 using electronic libraries in Scopus, PubMed, and EMBASE and manual exploration of the reference list of articles were the backbones of the current meta-analysis.” This is an ambiguous long sentence and shall be rephrased as “Our search was conducted on October 10, 2022, using electronic libraries in Scopus, PubMed, and EMBASE, as well as manual exploration of the reference lists of articles.”

Page 5, lines 122-124: “The study was cross-sectional, English-language full-text papers, the subject of study should be any type of university student, and studies should be done in Ethiopia.” This sentence needs grammatical revision.

Page, lines 124-127: “Studies published as review articles, qualitative studies, brief reports, letters to the editor, or editorial comments, working papers publications, published in a language other than English, research on non-human subjects, and studies with duplicate data from other studies were also excluded.” Why have you excluded qualitative inquiries while they are important in providing in-depth exploration of the individual students' experiences that could make your systematic review strong evidence to assure the prevalence of depression among your population of interest? Furthermore, why did you use research on non-human subjects as an exclusion criterion when your population of interest was students, i.e., human beings? This is totally out of the scope of your review, so it does not sound like it used animals as an exclusion criterion. The exclusion should be decided within the context of the research goal rather than on the basis of completely separate issues. By the way, could you list those listed as excluded article types?

Page 5, lines 127-128: “The PRISMA (preferred reporting items for systematic reviews and meta-analyses) standard was used to perform the literature search (36).” This is not clear. How did you use the PRISMA checklist for the literature search? As far as I know, they are used to frame the whole review process. The other major issue here is that the PRISMA version you cited is the 2009 version, but you used the PRISMA 2015 version in the supporting file. Why has the discrepancy taken place?

Page 5, lines 130-131: “Two authors (TA, and YZ) checked study titles and abstracts for eligibility after deleting duplicates.” How were the duplicates removed? For example, what automated tool(s) were used to remove the duplicates?

Page 5, lines 131-132: “The full texts were evaluated by the same reviewers if at least one of them thought an article was potentially eligible.” This is also not clear. Why did two authors participate or take part if one of them could decide without consensus?

Page 6, lines 148-149: What is the need to measure the Cochran Q-statistics if I^2^-indices are employed? Additionally, do you think that these statistics can provide information on the specific factor causing heterogeneity? You said that if I^2^ is zero, it shows the absence of heterogeneity. Did you think that you were correct or right? If you think so, this is absolutely unacceptable in statistics. Even the included studies have heterogeneity within themselves.

Page 6, lines 150-151: “Subgroup analysis and sensitivity analysis analyses were also used to investigate the source of heterogeneity among the studies included.” This sentence should be rewritten as "Subgroup and sensitivity analyses were also used to investigate the source of heterogeneity among the studies included."

Page 6, line 152: When do you think publication bias occurs?

**Results**

Page 7, line 166: “…5 articles were obtained from the reference list of included articles…” Could you please cite those articles? It is also recommended to spell out numbers with one digit rather than write them with their digit values.

Page 7, line 169: “…repetitive publications…” How many of them were published repeatedly? Please cite those resources.

Page 7, line 171: “…10 articles were tailored in the final meta-analysis…” How many articles were first included in the qualitative synthesis (the systematic review) before considering eligibility for the quantitative meta-analysis?

Page 7, lines 172-173: “…7 articles were poor methodological assessment, 4 articles were reviewed studies, and 2 articles were published other than the English language).” Please cite them.

Page 8, Table 1: "Tamene et al., 2021" is listed under the heading of the first column in an inappropriate place.

"Seid et al. 2019" is listed as reference number 33. However, even though the source is correct, the reference number 33 is listed differently as "Muhammed et al."

"Berhanu et al. 2020," which is listed in tables 1 and 2 and figures 2–5, is differently cited in reference list number 31.

"Teshome et al. 2019," which is listed as reference 30, is not consistent with its citation; for instance, the year is 2020 in the reference list.

Page 9, lines 202-204: “The pooled prevalence of depressive symptoms among university students from ten studies (28-35, 43, 44) included studies conducted in Ethiopia was 28.13% (95% CI: 22.67, 33.59) with significant heterogeneity among the studies (I2 = 100%, p-value < 0.001) (Fig 2).” It needs grammar revision.

Page 10, lines 230-231: “However, the result showed that there was no single influential study since the 95% CI interval result was obtained when each of the ten studies was excluded at a time (Fig 5).” It is not clear.

Page 12, lines 161-162: Please avoid the use of words such as "furthermore" and "also” simultaneously.

**Discussion**

Please do not repeat strategies, and procedures under this section, which you mentioned in the method’s section. Simply discuss your findings by comparing them to other relevant resources. For instance, the explanation from lines 274–282 is unnecessary.

Page 13, lines 268-272: This shall be discussed under the discussion's sub-title, "practical implications."

Page 14, lines 288-292: Is it appropriate to use "additionally" with "also" or "furthermore" with "also" simultaneously?

Page 14, line 294: “The findings revealed that several distinct characteristics…” Which findings? Yours or the comparators you mentioned? It is not clear.

Page 15, line 319: “…were were…” Please delete one.

Page 16, lines 344-347: Where did you get this? It needs to be cited. Or you should not be sure if it is your own justification.

**Strengths and limitations**

Page 16, line 349: Write "MA" in its expanded form, or you can abbreviate it from the beginning.

Can including old references be a strength of your review while our world is being drastically changed by technology, which is also a major reason for students to be frustrated with their education, which could result in anxiety and depression during exam time, and so on?

Please include other limitations such as pooling data despite high heterogeneity, studies other than cross-sectional, studies published in other languages, etc. Because all of these are the limitations of this review.

**Implications of this study for clinical practice, researchers, and policymakers**

Why did you skip the role of psychologists, psychiatrists, sociologists, lecturers, student counselors, etc. working in universities, as they are not stakeholders to take part in solving the issue?

**Conclusion**

Please be focused on the prevalence and the associated factors. Avoid making recommendations in this section, as they have already been mentioned under the implications section.

**Declarations**

Is the review protocol not registered in a registry data base, such as PROSPERO? If not, how did you know your review is the first of its kind in Ethiopia?

**References**

There are several major flaws within the organization of the reference lists. So, you should revise cautiously.
