# Supplementary material for: Prevalence of depression among students at Ethiopian universities and associated factors: A systematic review and meta-analysis
Source: PLoS One. 2023 Oct 12;18(10):e0288597. doi: 10.1371/journal.pone.0288597 (PMC10569578; doi:10.1371/journal.pone.0288597)
Supplement: Attachment — Submitted filename: Comments to the author.docx [file pone.0288597.s004.docx]

An important topic was raised in this review and meta-analysis even though meta-analysis in prevalence studies are hard to interpret and generalize because of considerable heterogeneity. I presented here my comments which helped the authors to strengthen their manuscript.

**Abstract**

**Result:** Change the sentence data was extracted…. to data were extracted…

Studies that utilized other (PHQ-9, HADS); 30.67% higher than studies that used BDI-II; 26.07 %. Not clear please reorganize this sentence.

Change aOR to AOR

**Conclusion:** change Ethipisn to Ethiopia

**Background**

Paragraph 1: guilty feeling repeated twice

Paragraph 3: Pakistani to Pakistan

Paragraph 4: In the context of Ethiopia too….not clear please reconstruct the sentence

**Materials and method**

It seems as you followed PRISMA checklist for reporting this systematic review and meta-analysis but you did not followed it correctly. Please follow The PRISIMA checklist while reporting and the whole method should be reorganized based on PRISMA checklist recommendation.

Please state the searching period correctly from when to when. You write a specific time period i.e. 10th of the October 2022.

In your inclusion and exclusion criteria you stated as studies ………published in a language other than English were excluded. Do you believe that studies conducted other than English language can be conducted and published in Ethiopia? I think there is no study conducted in any other language so please remove this.

The PRISMA (preferred reporting items for systematic reviews and meta-analyses) standard was used to perform the literature search. This sentence should be removed from this and written as a study protocol before search strategy.

You extract the detailed data using a standardized spreadsheet, what are the detailed data’s. It should be listed.

Since all the studies included for this systematic review and meta-analysis were crossectional studies, you will be used Newcastle Ottawa quality assessment scale adapted for crossectional studies to assess the quality of the studies rather than Newcastle Ottawa quality evaluation checklist. So you should to assess the quality of each study based on Newcastle Ottawa quality assessment scale adapted for crossectional studies again.

What is your outcome measurement? The outcome measurement should be explicitly stated before Data synthesis and analysis.

**Data analysis**

You stated as you used Higgs I^2^ statistic and Cochran Q-statistics were also utilized to detect heterogeneity. But you did not use Cochran Q-statistics? Why?

The I^2^ statistical values of zero, 25, 50, and 75% connote absence, little, moderate, and great heterogeneity, respectively. This is vague please categorize it.

**Results**

Your systematic review and meta-analysis is conducted in Ethiopia and your search will be studies conducted in Ethiopia. How 2 articles were removed because published other than the English language.

There is a considerable heterogeneity I^2^ =100%, so you tried to explain the source of heterogeneity using subgroup analysis considering the sample size of each study and the study instrument. Even the source of heterogeneity is not explained, I^2^ =100%. If the source of heterogeneity is not explained, what you would do? Why you will limit subgroup analysis on sample size and the study instrument
