# Supplementary material for: Prevalence of depression among students at Ethiopian universities and associated factors: A systematic review and meta-analysis
Source: PLoS One. 2023 Oct 12;18(10):e0288597. doi: 10.1371/journal.pone.0288597 (PMC10569578; doi:10.1371/journal.pone.0288597)
Supplement: Attachment — Submitted filename: Response to Reviewers.docx [file pone.0288597.s006.docx]

**Comments to the authors**

**PLOS ONE**

PONE-D-22-34689

Article Type: Study Protocol

Title: Prevalence and associated factors of depressive symptoms among Ethiopian University students: a systematic review and meta-analysis

Thank you dear Editor, and Reviewers for your great contribution. We will address the following issues ;

1. Avoid Email addresses except the correspondence author in the title page.

Tamrat Anbesaw, Wollo University, College of Medicine and Health 26 Sciences, Department of Psychiatry, Dessie, Ethiopia, should be revised as “*Corresponding author”.

**Response:** We removed it.

2. Authors’ contributions should be prepared based on the journal guideline.

**Response:** We formatted the manuscript using the PLoS ONE style.

**Authors contributions**

Conceptualization: Tamrat Anbesaw, Yosef Zenebe.

Data curation: Tamrat Anbesaw, Yosef Zenebe, Mogessie Necho,Tesfaye Segon.
Formal analysis: Tamrat Anbesaw, Yosef Zenebe

Funding acquisition: Tamrat Anbesaw, Yosef Zenebe.
Investigation: Tamrat Anbesaw, Yosef Zenebe.

Methodology: Tamrat Anbesaw, Yosef Zenebe, Mogessie Necho, Moges Gebresellassie,Tesfaye Segon^,^ Fasikaw Kebede, Tilahun Bete
Project administration: Tamrat Anbesaw, Yosef Zenebe

Resources: Tamrat Anbesaw, Yosef Zenebe, Mogessie Necho, Moges Gebresellassie,Tesfaye Segon, Tilahun Bete
Software: Tamrat Anbesaw, Yosef Zenebe

Validation: Tamrat Anbesaw, Yosef Zenebe
Writing – original draft: Tamrat Anbesaw

Writing – review & editing: Tamrat Anbesaw, Yosef Zenebe, Mogessie Necho, Tesfaye Segon^,^ Fasikaw Kebede, Tilahun Bete
